# Nurse-to-family telehealth for pediatric transfers: protocol for a feasibility and pilot cluster randomized controlled trial

**DOI:** 10.1186/s40814-023-01292-4

**Published:** 2023-04-11

**Authors:** Jennifer L. Rosenthal, Adrienne E. Hoyt-Austin, Audriana Ketchersid, April Sanders, Thomas A. Harper, Daniel J. Tancredi, Heather M. Young, Patrick S. Romano, James P. Marcin

**Affiliations:** 1 Department of Pediatrics, University of California Davis, 2516 Stockton Blvd, Sacramento, CA 95817 USA; 2 Center for Health and Technology, University of California Davis, 4610 X Street, Sacramento, CA 95817 USA; 3 Betty Irene Moore School of Nursing, University of California Davis, 2570 48Th St., Sacramento, CA 95817 USA; 4 Department of Internal Medicine and Center for Healthcare Policy and Research, University of California Davis, 4150 V St., Sacramento, CA 95817 USA

**Keywords:** Pediatrics, Clinical trial, Feasibility study, Pilot study, Telemedicine, Hospital medicine, Emergency medicine, Patient transfer, Patient-centered care, Nurses

## Abstract

**Background:**

Children presenting to emergency departments of community hospitals may require transfer to a children’s hospital for more definitive care, but the transfer process can be distressing and burdensome to patients, families, and the healthcare system. Using telehealth to bring the children’s hospital nurse virtually to the bedside of the child in the emergency department has the potential to promote family-centered care and minimize triage issues and other transfer-associated burdens. To explore the feasibility of the nurse-to-family telehealth intervention, we are conducting a pilot study.

**Methods:**

This parallel cluster randomized controlled feasibility and pilot trial will randomize six community emergency departments to use either nurse-to-family telehealth (intervention) or usual care (control) for pediatric inter-facility transfers. All eligible children presenting to a participating site during the study period who require inter-facility transfer will be included. Eligibility requires that there be an English-speaking adult parent or guardian at the emergency department bedside. We will examine feasibility objectives that assess protocol assignment adherence, fidelity, and survey response rates. We will measure subject-level exploratory outcome data to test feasibility of data collection and to obtain effect size estimates; exploratory outcomes include family-centered care, family experience, parent acute stress, parent distress, and change in level of care. Additionally, we will conduct a mixed methods implementation evaluation using the RE-AIM (Reach, Effectiveness, Adoption, Implementation, Maintenance) framework.

**Discussion:**

The findings from this trial will increase our understanding about nurse-to-family telehealth during pediatric transfers. The mixed methods implementation evaluation will provide relevant insight about the contextual factors that influence the implementation and rigorous evaluation of our intervention.

**Trial registration:**

ClinicalTrials.gov Identifier: NCT05593900. First Posted: October 26, 2022. Last Update Posted: December 5, 2022.

## Background

Children who present to the emergency department (ED) of a hospital lacking needed pediatric resources [1–3] may require a transfer to a specialty hospital for definitive care. Nearly 350,000 children are transferred annually, and this number is rising over time [4]. Although these transfers are often lifesaving, some transfers cause preventable harm to the child and their families due to poor communication and lack of family-centered care [5]. Specifically, parents or guardians (referred to as “parents” hereafter) can experience emotional stress, cost burdens, and distrust [5].

Key components of delivering patient- and family-centered care include effective communication, collaboration, empowerment, respect, individualized care, and support [6, 7]. Providing family-centered care is associated with improved care quality, greater trust, reduced parent anxiety, better parent experience, and reduced cost burdens [7–9]. However, important barriers prevent families from consistently experiencing family-centered care, including family and provider stress, competing demands, and organizational limitations [10, 11]. The circumstances of a pediatric ED-to-hospital transfer intensify these challenges, exposing parents to anxiety, distrust, and uncertainty [5, 12, 13]. Children can also be transferred to an intensive care unit (ICU) when they do not require that level of care [14]. This secondary over-triage imposes additional stress and burden on patients, parents, and the healthcare system.

The use of telehealth to bring a pediatric provider virtually to the child’s bedside in the ED has the potential to promote more family-centered care during transfers and to mitigate distress and triage issues [5, 14–18]. In this protocol report, telehealth refers to information exchange from one site to another using HIPAA-compliant videoconference technology [19]. Telehealth use may increase family-centeredness of care by improving communication, engagement, and coordination of care [20].

Most pediatric inter-facility telehealth research has examined the use of telehealth by physicians [14–17]. The research examining telehealth use by nurses has studied nurse-to-nurse telehealth handoffs during the transfer process [21]. However, the feasibility and impact of nurse-to-family telehealth visits during pediatric transfers remains a research gap. Testing nurse-to- family telehealth visits is a promising approach, because research suggests that the information most parents seek during a hospital transfer relates to preparing them for what to expect at the post-transfer hospital and includes information that a registered nurse, rather than a physician, is well-positioned to communicate [22]. Additionally, an early nurse assessment can identify necessary clinical needs to better prepare for the patient’s arrival.

Our central hypothesis is that telehealth use to connect a care team member from the receiving hospital virtually with the child’s parents may increase family-centeredness of care, reduce parent stress, and improve triage appropriateness. A pilot study is needed to explore the feasibility of conducting a nurse-to-family telehealth trial. Pilot studies are most effective when they explore study logistics (e.g., adoption, fidelity, feasibility of data collection) and obtain empirical evidence of study parameters to inform the eventual definitive study [23–25]. We therefore aim to test the feasibility of conducting a parallel cluster randomized trial comparing nurse-to-family telehealth communication to usual care for pediatric transfers.

## Methods

This trial protocol follows the SPIRIT (Standard Protocol Items: Recommendations for Interventional Trials) guideline [26]. The reporting of this protocol incorporates elements from the CONSORT extension to pilot and feasibility trials [27].

### Trial design

**Fig. 1:**
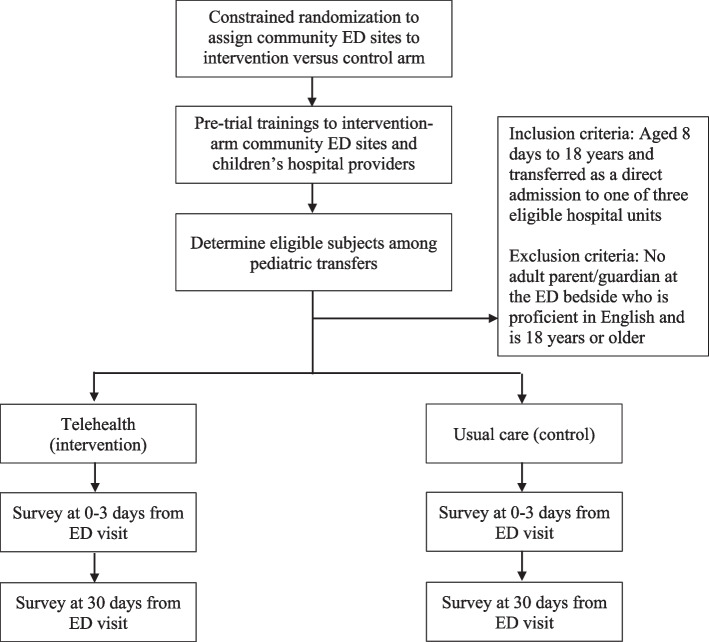
Overview of the trial procedures. Legend: ED—emergency department

This pilot study will use a parallel cluster randomized controlled trial design. Figure 1 shows the overview of the trial procedures. We will examine feasibility objectives and exploratory outcomes. Additionally, we will conduct a mixed methods implementation evaluation using the RE-AIM (Reach, Effectiveness, Adoption, Implementation, Maintenance) framework [28] to understand how to optimize the translation of our intervention across diverse groups and settings [29]. Early attention to implementation outcomes (i.e., during pilot testing) is a valuable strategy to facilitate the translation of research into practice.

### Setting and population

The post-transfer hospital is a metropolitan tertiary care children’s hospital within a university hospital. The pre-transfer sites include six community hospital EDs that are located from 9 to 160 miles away, both rural and suburban. These sites all have inpatient pediatric capabilities; therefore, the children transferred to the children’s hospital need specialty care and/or higher levels of service.

Eligible patients will be children aged eight days to less than or equal to 18 years who present to a participating community hospital ED, are accepted for inter-facility transfer, are assigned to arrive as a direct admission (i.e., ED-to-inpatient) to one of three eligible hospital units, and have an adult parent or guardian at the bedside with English proficiency. The eligible units include the pediatric ICU and two pediatric acute care units. Transfer consultations for children less than 8 days of age and those destined for the neonatal ICU will be excluded, since neonatal patients have unique transfer patterns [30, 31]. Although pediatric patients are sometimes directly admitted to adult units, we limit the eligible units to the three primary pediatric units to support feasibility of training care team members in this pilot trial. Likewise, we limit this trial to participants who are directly admitted—excluding ED-ED transfers—to prioritize feasibility and first test the intervention in a homogeneous context. This trial is limited to parents with English proficiency, because we will use an existing telehealth platform that is only available in English. We will first pilot test this intervention before adapting the platform’s family-facing interface into additional languages.

All eligible children will be enrolled in the study. This research has a waiver of consent for the intervention, because telehealth visits are an existing clinical resource that can be used for all transferring patients. The decision to use telehealth in this trial will be encouraged in the intervention arm but will remain optional; no nurse or parent will be required to use telehealth. The parent survey that will be used for data collection of exploratory outcomes will have elements of informed consent before the survey questions.

### Feasibility objectives

Feasibility will be assessed by protocol assignment adherence, fidelity, and survey response rates. Table 1 shows the three feasibility objective indicators and established criteria for success. Feasibility objective 1 (primary feasibility objective) will be assessed by tracking adherence, defined as the proportion of eligible patients for whom the protocol assignment (telehealth initiated versus usual care [no telehealth]) is followed. Fidelity rates for objective 2 will be measured using a dichotomous rating of whether the telehealth connection is initiated prior to the patients’ arrival to the children’s hospital. Objective 3 will be assessed based on survey response rates for each survey-derived outcome: family-centered care, family experience, parent acute stress, and parent distress.

**Table 1:** Feasibility objectives

Feasibility objective	Indicator	Criteria for success
(1) Protocol assignment adherence	Proportion of eligible patients for whom the protocol assignment (telehealth initiated versus usual care) is followed	Adherence will be 75% or greater
(2) Fidelity: timing of delivery	Whether the telehealth connection is initiated prior to the patients’ arrival to the children’s hospital	Fidelity will be 75% or greater
(3) Survey response rate	Survey response rates for each survey-derived outcome	Survey response rates will be 75% or greater

### Exploratory outcomes

We will measure subject-level exploratory outcomes data to test feasibility of data collection and to obtain effect size estimates. Exploratory outcomes include family-centered care, family experience, parent acute stress, parent distress, and change in level of care. Family-centered care of the community ED encounter will be assessed using the ED Family-Centered Care Experience (ED-FACCE) survey [32]. Family experience will be measured using the two items measuring overall experience from the ED Consumer Assessment of Healthcare Providers and Systems (CAHPS) survey [33]. Parent acute stress and distress will be assessed using the Emotional Distress: Anxiety measure [34]. Change in level of care will be measured at two timepoints: (1) between initial unit assignment and post-transfer arrival and (2) between post-transfer arrival until 12 h after arrival. Change in level of care will be defined as switching from an acute care unit or service to an intensive care unit or service, and vice versa.

### Study procedures

#### Randomization

The unit of randomization will be the community hospital, which decreases contamination in comparison to randomization at the patient level (under which each ED would have simultaneous telehealth visits and usual care). We will use constrained randomization to better balance the intervention and control arms with respect to ED-to-children’s hospital distance and pediatric transfer volume. Distance will be categorized into less than 21 miles, 21–50 miles, and more than 50 miles. Transfer volume will be categorized into less than 21, 21–60, and more than 60 annual pediatric transfers that arrive as direct admissions to a pediatric ICU or pediatric acute care unit.

#### Intervention

Per standard procedures, after a children’s hospital physician accepts a patient for transfer, the transferring patient is assigned a hospital unit by the bed control nurse. The charge nurse will accept or deny the patient to their unit. Upon accepting a patient to their unit, this charge nurse will determine whether the transferring patient meets trial eligibility criteria and is transferring from an intervention-arm ED site. Upon confirmation that the patient is eligible to receive a nurse-to-family telehealth visit, the charge nurse will call by telephone the community hospital ED and ask the bedside nurse to offer a telehealth visit to the patient’s parent(s). If the parents would like to have a nurse-to-family telehealth visit, the ED nurse will give the parent’s cell phone number or email address to the children’s hospital charge nurse.

The children’s hospital charge nurse will then use their video-enabled computer to launch a telehealth visit using the secure telehealth application called ExtendedCare. The ExtendedCare platform meets Health Insurance Portability and Accountability Act security rules and launches from the patient’s electronic health record. From within this telehealth visit, the charge nurse will send an electronic message (e.g., via text or email) to the parent and wait for the parent to join the visit (establish a secure videoconference). The message to the parent includes a link that can be clicked to open a browser that allows the parent to join the telehealth visit. The parent does not need to download or use any application or program.

Charge nurses are instructed to initiate the telehealth visit prior to the patient leaving the community hospital ED and to wait at least 5 min for the parent to join the telehealth connection. Charge nurses are also instructed that the content of the discussion during the visit is intended to prepare the family and charge nurse for the transfer process and the patient’s arrival. Suggested topics that can be discussed during the visit include visitation policies, sleeping arrangements, resources (e.g., food and milk storage), and the child’s special needs, if any. This workflow requires that the parent has a smart phone with access to text messaging or email. If a parent does not have a smart phone with access to text or email but would like to speak with the charge nurse, a phone call will be offered instead. Other interventions related to parent-provider communication that are delivered prior to the arrival at the post-transfer hospital will be prohibited during the trial.

#### Control

Subjects presenting to control-arm ED sites will receive usual care. These subjects will transfer to the children’s hospital without the parent communicating with the charge nurse. The charge nurse will not have direct communication with anyone from the ED. The charge nurse will communicate with the children’s hospital bed control nurse and review the written documentation about the patient in the electronic health record.

#### Data collection

Data collection for the feasibility objectives and exploratory outcomes will include chart review of the electronic health record and parent surveys. Parent surveys will be distributed to one parent per enrolled patient. Parent respondents will include only English-proficient individuals who were with their child during the ED visit. For children who had more than one parent at their ED bedside, we will let the parents select the single respondent. Eligible parents will receive the family-centered care, experience, and anxiety instruments 0–3 days after the ED encounter. Parent distress will be measured at 30 days from the ED encounter. Surveys not completed within 21 days of distribution will be considered non-response. Participants will receive a $25 gift card for each survey packet that they return. Parents completing both survey packets will therefore receive a total of $50 in gift cards.

Data collection for the exploratory outcome change in level of care will use chart review. Electronic health record data will be used to abstract patient demographics (age, race, ethnicity, sex, insurance, California Healthy Places Index [35]) and utilization variables (admitting service, length of stay, disposition). Demographic characteristics of family caregivers (age, race, ethnicity, gender, education, smart phone access, digital literacy score [36]) will be collected in the survey packets.

#### Analysis

For feasibility objectives, proportions with 95% CI will be calculated for protocol assignment adherence, fidelity, and survey response rates to determine if each of the feasibility objectives is met. For the exploratory outcomes, analyses will be descriptive, designed to provide effect size estimates with 95% CI. To account for the cluster randomized design, we will use analysis methods for clustered data when estimating confidence intervals, treating ED sites as clusters.

#### Sample size

Our cluster randomized design and analysis strategy will inflate our confidence intervals in two ways: (1) by the impact of intracluster correlation (ICC) on the variance of estimated means and proportions and (2) by the use of critical values from t-distributions whose degrees of freedom are based on the number of clusters, not the number of participants [37]. For Objectives 1 and 3, six sites will result in 120 participants. For Objective 2, the three intervention sites are expected to yield 45 participants for the fidelity testing. For our binomial outcomes, we conservatively assumed that the overall probability could be 0.50, the value maximizing its variance, and that 95% of the sites from a hypothetical superpopulation of sites—such as the ones in our study—would have true site-specific probabilities within 14 percentage points of the overall probability, which translates into a modest ICC of 2% [38]. Hence, applying Kish’s clustered data variance inflation factor (VIF) formula that describes how the variance of sample means from a study with clusters of average size m is affected by ICC (VIF = 1 + [m − 1]*ICC) [39], we anticipate clustered variance inflation factors of 1.38 for Objectives 1 and 3 and 1.28 for Objective 2.

The second sample size penalty from Cornfield for cluster randomized trials is the one that is needed to account for the smaller number of degrees of freedom. It is based on the ratio of the relevant quantiles for the test statistic sampling distributions when degrees of freedom are based on the number of clusters, not the number of participants. For t-statistic quantiles, this ratio needs to be squared for it to be able to quantify relative impacts on the effective sample size [40, 41]. For Objectives 1 and 3, the 97.5% quantiles from t-distributions with 5 and 119 degrees of freedom are approximately 2.57 and 1.98, respectively, resulting in a degrees-of-freedom penalty factor of approximately 1.68, the square of 2.57/1.98. For Objective 2, the degrees-of-freedom penalty factor is 4.56. Hence, for Objectives 1 and 3, the product of the two penalties is approximately 2.33, converting the actual sample size of 120 into an effective sample size of approximately 51, permitting us to estimate for our feasibility outcomes proportions that will have error margins < 14 percentage points. For Objective 2, an actual sample size of 45 results in an effective sample size of approximately 8, so that our fidelity outcome proportions will have error margins that may be as high as 40 percentage points and thus of little practical value [42].

This pilot trial is not powered to determine the relative benefit of telehealth versus usual care [23–25], as that important question will be answered in a future efficacy trial. The chosen sample size of 120 patient encounters prioritizes feasibility over power.

### Implementation evaluation

We will apply a pragmatic use of the five dimensions of the RE-AIM framework [28, 29] to conduct an intervention implementation evaluation within this pilot trial. We will use a mixed methods approach with a convergent design [43]. Table 2 shows how we will use quantitative and qualitative items relevant to each RE-AIM dimension.

**Table 2:** Quantitative and qualitative items for each RE-AIM dimension

Dimension	Quantitative items	Qualitative items
Reach	• % excluded and characteristics • Characteristics of parent(s)/child who received telehealth among intervention arm subjects	• Explore factors influencing reach
Effectiveness	• Between arm effect size estimates for exploratory outcomes	• Explore perceived benefits and challenges • Explore mechanisms of action for outcomes • Explore mechanisms of potential heterogeneity effects
Adoption	• % and characteristics of users vs. non-users among charge nurses with an eligible intervention arm subject	• Explore factors influencing parent participation • Explore factors influencing provider participation
Implementation	• Duration (minutes) of telehealth wait time and connection time	• Explore factors influencing implementation • Implementation adaptations made
Maintenance	• % per month of telehealth used for transfers post-trial	• Explore aspects sustained/modified post-trial

#### Quantitative phase

We will use descriptive statistics to summarize subject- and parent-level characteristics. We will analyze all available data. Any variables with 10% or greater missing data will be flagged. We will investigate the contributing factors that result in relatively high rates of missing data.

#### Qualitative phase

Qualitative data collection will include parent surveys and interviews. The previously described parent survey will include an optional free-text response question inviting parents to provide additional thoughts or feedback about their experience with their child’s transfer. We will also conduct in-depth interviews with a sample of adult parents and care team providers (e.g., nurses, unit clerks, social workers, physicians). Parents will include those from the trial intervention arm. We will begin with convenience sampling and then use purposive sampling [44] to ensure diversity of intervention use, role, and demographics. We will interview ~ 30 individuals, concluding when we reach thematic saturation. Parent recruitment will occur within 2 weeks of the hospital transfer. Provider recruitment will occur during the last 3 months of the trial. Providers will be recruited from the three community ED intervention-arm sites and the post-transfer children’s hospital. One-on-one interviews will last ~ 45 min. Interviews will be audio recorded, professionally transcribed and deidentified, and reviewed for accuracy. Interviewers will maintain notes with contextual observations and cues. Participants will receive a $50 gift card.

We will use thematic analysis. Four research team members will independently memo and code the initial three interview transcripts and ten survey free-text responses using a priori codes pertaining to the RE-AIM [28] dimensions while identifying emergent codes. We will then meet to discuss the coding structure and new topics from inductive coding. After this meeting, we will independently memo and code 2–5 transcripts and texts and meet again to discuss code application, refine and add codes, develop categories, and revise the interview guide. This iterative process will be repeated every 2–5 transcripts and texts. We will revisit prior transcripts as new codes are identified. We will identify linkages and patterns between the codes, which will become analytic themes. Once the data appear to coalesce around similar themes, we will conclude that data saturation is met. Finally, we will solicit interviewee respondent validation on the themes. We will use ATLAS.ti [45] to organize the data and maintain an audit trail using a team journal to document the qualitative procedures.

#### Integration

We will use a convergent design [43] to integrate the quantitative and qualitative data. We will compare quantitative and qualitative data using a matrix to relate the two types of data to each other and identify congruent and divergent results. Should there be discrepancies between the quantitative and qualitative findings, we will reexamine the existing databases to gain additional insight and attempt to resolve the discrepancies [43]. Should discrepancies remain that require further inquiry, we will adapt the mixed methods approach to become multiphase and conduct additional interviews to explore these discrepancies. We will report the merged data using narrative integration and joint display.

### Stakeholder engagement

This protocol was developed in collaboration with a stakeholder team comprised of four parents, seven nurses, seven physicians, and four telehealth or information technology staff. The engagement plan to develop the protocol included one-on-one interviews, a human-centered design workshop, group feedback sessions, and one-on-one feedback sessions. Engagement sessions were in person when possible and otherwise via videoconference. Pre-implementation engagement outcomes included refinement of the intervention procedures (e.g., workflows), documents (e.g., training materials), and exploratory outcome measures.

We will continue to engage stakeholders throughout the trial process to conduct a relevant, acceptable, and effective intervention study [46]. Individual stakeholders will assist with trainings at their respective sites during the pre-trial preparation phase. They will assist with site visits during the intervention period. They will also assist in troubleshooting challenges that arise, such as low protocol assignment adherence rates, fidelity rates, or survey response rates. We will have a mid-trial team check-in to solicit perceptions, experiences, and input. At the conclusion of the trial, we will have another team session to solicit stakeholder perspectives on data interpretation, how to disseminate the trial findings, and future directions. Team sessions will occur via videoconference to overcome transportation barriers and other challenges for our stakeholders. Stakeholders receive $30/h [47] in gift cards for the team sessions.

### Monitoring and dissemination

This study involves no more than minimal risk. A Data Safety Monitoring Plan will be used for this study as a protection measure per the requirements of a clinical trial. An Independent Monitoring Committee will be convened to assess the progress of this pilot trial and the safety data. The committee members will consist of three pediatricians not associated with the study. The Independent Monitoring Committee will review cumulative study data to evaluate safety, study conduct, validity, trial conduct, and data integrity. The committee will meet to independently review outcomes on a quarterly basis and as needed based on any reported complications. The safety monitoring will begin when the trial enrollment begins. The committee will complete quarterly reports detailing the study progress, any adverse events, and any protocol deviations. Interim statistical analysis will not be performed for this pilot trial that is primarily descriptive and focused on feasibility.

## Discussion

This feasibility and pilot trial will increase our understanding about nurse-to-family telehealth use during pediatric transfers. This trial is designed to provide evidence of whether to proceed with a subsequent definitive efficacy trial. If the feasibility objectives are achieved, we will have sufficient evidence to support a future larger randomized controlled trial to examine the efficacy of nurse-to-family telehealth use for pediatric transfers. Our trial team will also consider the implementation evaluation findings and additional external factors.

The mixed methods RE-AIM implementation evaluation will provide relevant insight about the contextual factors that influence implementation of our intervention. We need telehealth solutions that increase equitable access to care rather than worsen disparities [48]. Underserved populations have disproportionately lower access to telehealth services [49–51]. Research that evaluates the issues and dimensions that impact the reach and adoption of telehealth among diverse groups is critically needed [52, 53]. Applying the RE-AIM framework to evaluate telehealth interventions is a strategy to address this need, as RE-AIM explicitly focuses on these design and implementation processes [29]. Incorporating implementation trial elements into clinical trials is a strategy that can mitigate the research-to-practice translation gap [54]. Lessons learned from our mixed methods implementation evaluation will be used to refine the future trial design.

## Data Availability

The datasets used and/or analyzed during the current study are available from the corresponding author on reasonable request.

## Abbreviations

ED: Emergency department
ICU: Intensive care unit
RE-AIM: Reach, Effectiveness, Adoption, Implementation, Maintenance
ICC: Intracluster correlation

## References

[1] Middleton KR, Burt CW. Availability of pediatric services and equipment in emergency departments: United States, 2002-03. Adv Data. 2006;(367):1–16. 16544808

[2] Schappert SM, Bhuiya F (2012). Availability of pediatric services and equipment in emergency departments: United States, 2006. Natl Health Stat Report.

[3] Institute of Medicine, Board on Health Care Services, Committee on the Future of Emergency Care in the United States Health System (2007). Emergency care for children: growing pains.

[4] França UL, McManus ML. Trends in Regionalization of Hospital Care for Common Pediatric Conditions. Pediatrics. 2018;141(1):e20171940. 10.1542/peds.2017-1940. 10.1542/peds.2017-1940 29263253

[5] Rosenthal JL, Li S-TT, Hernandez L, Alvarez M, Rehm RS, Okumura MJ (2017). Familial caregiver and physician perceptions of the family-physician interactions during interfacility transfers. Hospital Pediatr.

[6] Committee on Hospital Care and Institute for Patient- and Family-Centered Care (2012). Patient-and family-centered care and the pediatrician's role. Pediatrics.

[7] Coyne I, Holmström I, Söderbäck M (2018). Centeredness in healthcare: a concept synthesis of family-centered care, person-centered care and child-centered care. J Pediatr Nurs.

[8] Kuo DZ, Houtrow AJ, Arango P, Kuhlthau KA, Simmons JM, Neff JM (2012). Family-centered care: current applications and future directions in pediatric health care. Matern Child Health J.

[9] Goldfarb MJ, Bibas L, Bartlett V, Jones H, Khan N (2017). Outcomes of patient-and family-centered care interventions in the ICU: a systematic review and meta-analysis. Crit Care Med.

[10] Mirlashari J, Brown H, Fomani FK, de Salaberry J, Zadeh TK, Khoshkhou F (2020). The challenges of implementing family-centered care in NICU from the perspectives of physicians and nurses. J Pediatr Nurs.

[11] Oude Maatman SM, Bohlin K, Lilliesköld S (2020). Factors influencing implementation of family-centered care in a neonatal intensive care unit. Front Pediatr.

[12] Rosenthal JL, Perez SL, Young HM (2023). Contextual factors influencing parents' assessments of family-centred care in the paediatric emergency department: a qualitative study. Nurs Open.

[13] Sauers-Ford HS, Aboagye JB, Henderson S, Marcin JP, Rosenthal JL (2021). Disconnection in information exchange during pediatric trauma transfers: a qualitative study. J Patient Exp.

[14] Harvey JB, Yeager BE, Cramer C, Wheeler D, David MS (2017). The impact of telemedicine on pediatric critical care triage. Pediatr Crit Care Med.

[15] Desai S, Williams ML, Smith AC (2013). Teleconsultation from a secondary hospital for paediatric emergencies occurring at rural hospitals in Queensland. J Telemed Telecare.

[16] Tachakra S, Uche CU, Stinson A (2002). Four years' experience of telemedicine support of a minor accident and treatment service. J Telemed Telecare.

[17] Marcin JP, Nesbitt TS, Struve S, Traugott C, Dimand RJ (2004). Financial benefits of a pediatric intensive care unit-based telemedicine program to a rural adult intensive care unit: impact of keeping acutely ill and injured children in their local community. Telemed J E Health.

[18] Sauers-Ford HS, Hamline MY, Gosdin MM (2019). Acceptability, usability, and effectiveness: a qualitative study evaluating a pediatric telemedicine program. Acad Emerg Med.

[19] American Telemedicine Association. Telemedicine glossary. Available at: http://hub.americantelemed.org/resources/telemedicine-glossary. Accessed 5 Oct 2016.

[20] Byczkowski TL, Gillespie GL, Kennebeck SS, Fitzgerald MR, Downing KA, Alessandrini EA (2016). Family-centered pediatric emergency care: a framework for measuring what parents want and value. Acad Pediatr.

[21] Lieng M, Siefkes H, Sauers-Ford H (2019). Telemedicine for interfacility nurse handoffs. Ped Critical Care.

[22] Rosenthal JL, Haynes SC, Bonilla B (2022). Enhancing the implementation of the virtual pediatric trauma center using practical, robust, implementation and sustainability model: a mixed-methods study. Telemed Rep.

[23] Kistin C, Silverstein M (2015). Pilot studies: a critical but potentially misused component of interventional research. JAMA.

[24] Leon AC, Davis LL, Kraemer HC (2011). The role and interpretation of pilot studies in clinical research. J Psychiatr Res.

[25] Moore CG, Carter RE, Nietert PJ, Stewart PW (2011). Recommendations for planning pilot studies in clinical and translational research. Clin Transl Sci.

[26] Chan A-W, Tetzlaff JM, Altman DG (2013). SPIRIT 2013 statement: defining standard protocol items for clinical trials. Ann Intern Med.

[27] Eldridge SM, Chan CL, Campbell MJ (2016). CONSORT 2010 statement: extension to randomised pilot and feasibility trials. BMJ.

[28] Glasgow RE, Vogt TM, Boles SM (1999). Evaluating the public health impact of health promotion interventions: the RE-AIM framework. Am J Public Health.

[29] Glasgow RE, Harden SM, Gaglio B (2019). RE-AIM planning and evaluation framework: adapting to new science and practice with a 20-year review. Front Public Health.

[30] Rosenthal JL, Hilton JF, Teufel RJ, Romano PS, Kaiser SV, Okumura MJ (2016). Profiling interfacility transfers for hospitalized pediatric patients. Hosp Pediatr.

[31] Huang Y, Natale JE, Kissee JL, Dayal P, Rosenthal JL, Marcin JP (2017). The association between insurance and transfer of noninjured children from Emergency Departments. Ann Emerg Med.

[32] Rosenthal JL, Albano AD, Tancredi DJ, Perez SL, Young HM, Romano PS (2022). Development and psychometric evaluation of a caregiver survey to assess family-centered care in the emergency department. Acad Pediatr.

[33] Weinick RM, Becker K, Parast L (2014). Emergency department patient experience of care survey: development and field test. Rand Health Q.

[34] Pilkonis PA, Choi SW, Reise SP (2011). Item banks for measuring emotional distress from the Patient-Reported Outcomes Measurement Information System (PROMIS®): depression, anxiety, and anger. Assessment.

[35] Public Health Alliance of Southern California. Healthy places index. Available at: https://www.healthyplacesindex.org. Accessed 3 Nov 2022.

[36] Nelson LA, Pennings JS, Sommer EC, Popescu F, Barkin SL (2022). A 3-item measure of digital health care literacy: development and validation study. JMIR Form Res.

[37] Cornfield J (1978). Symposium on CHD prevention trials: design issues in testing life style intervention: randomization by group: a formal analysis. Am J Epidemiol.

[38] Hayes RJ, Moulton LH. Cluster randomised trials, second edition. CRC Press. 2017. 10.4324/9781315370286.

[39] Kish L. Survey sampling. New York: Wiley; 1965.

[40] Franco C, Little RJ, Louis TA, Slud EV (2019). Comparative study of confidence intervals for proportions in complex sample surveys. J Surv Stat Methodol.

[41] Korn EL, Graubard BI. Analysis of Health Surveys. Wiley Series in Probability and Statistics Survey Methodology Section. New York: Wiley; 1999. 10.1002/9781118032619.

[42] Billingham SA, Whitehead AL, Julious SA (2013). An audit of sample sizes for pilot and feasibility trials being undertaken in the United Kingdom registered in the United Kingdom Clinical Research Network database. BMC Med Res Methodol.

[43] Creswell JW, Clark VLP (2017). Designing and conducting mixed methods research.

[44] Tongco MDC (2007). Purposive sampling as a tool for informant selection. Ethnobot Res Appl.

[45] ATLAS.ti Scientific Software Development GmbH [ATLAS.ti 22 Mac]. 2022. Retrieved from https://atlasti.com. https://atlasti.com/research-hub/citing-atlas-ti-in-your-research.

[46] Fleurence RL, Curtis LH, Califf RM, Platt R, Selby JV, Brown JS (2014). Launching PCORnet, a national patient-centered clinical research network. J Am Med Inform Assoc.

[47] Independent Sector. Value of volunteer time. Available at: http://www.independentsector.org/volunteer_time. Accessed 21 Aug 2018.

[48] Westby A, Nissly T, Gieseker R, Timmins K, Justesen K (2021). Achieving equity in telehealth: “centering at the margins” in access, provision, and reimbursement. J Am Board Fam Med.

[49] Eberly LA, Khatana SAM, Nathan AS (2020). Telemedicine outpatient cardiovascular care during the COVID-19 pandemic: bridging or opening the digital divide?. Circulation.

[50] Blundell AR, Kroshinsky D, Hawryluk EB, Das S (2021). Disparities in telemedicine access for Spanish-speaking patients during the COVID-19 crisis. Pediatr Dermatol.

[51] Roberts ET, Mehrotra A (2020). Assessment of disparities in digital access among medicare beneficiaries and implications for telemedicine. JAMA Intern Med.

[52] Wetsman, N. Telehealth wasn’t designed for non-English speakers. Available at https://www.theverge.com/21277936/telehealth-english-systems-disparities-interpreters-online-doctor-appointments. Accessed 5 Mar 2020.

[53] UCLA Latino Policy and Politics Initiative. Telehealth and COVID-19: policy considerations to improve access to care. Available at: https://latino.ucla.edu/research/telehealth-covid-19-policy-considerations-to-improve-access-to-care/. Accessed 15Aug 2021.

[54] Curran GM, Bauer M, Mittman B, Pyne JM, Stetler C (2012). Effectiveness-implementation hybrid designs: combining elements of clinical effectiveness and implementation research to enhance public health impact. Med Care.

